# Possibility of decryption speed-up by parallel processing in CCA secure hashed ElGamal

**DOI:** 10.1371/journal.pone.0294840

**Published:** 2023-11-30

**Authors:** Gyu Chol Kim, Hyon A. Ji, Yong Bok Jong, Gwang Hyok Kim, Hak Su Kim

**Affiliations:** Faculty of Information Science and Technology, Kim Chaek University of Technology, Pyong Yang, Democratic People’s Republic of Korea; Jaramogi Oginga Odinga University of Science and Technology, KENYA

## Abstract

In order to prove the ElGamal CCA(Chosen Ciphertext Attack) security in the random oracle model, it is necessary to use the group where ICDH(Interactive Computational Diffie Hellman) assumption holds. Until now, only bilinear group with complex algebraic structure has been known as the ICDH group. In this paper, we introduce the ICDH group with simple algebraic structure. In other words, we prove that ICDH assumption holds in the integer group with composite modulus. On the basis of this, we propose the CCA secure hashed ElGamal and its fast variant to speed up decryption by parallel processing. Our parallel scheme has the fastest decryption among all CCA secure PKE(Public Key Encryption) schemes implemented in integer group and gives the possibility that ElGamal protocol could be practical when the big modulus numbers are used to resist the quantum attack.

## 1. Introduction

After the discovery of DH(Diffie Hellman) key exchange protocol [1], many PKE schemes [2–8] based on CDH(Computational Diffie Hellman) problem have been developed and widely used. In modern ElGamal systems, using DH value itself to mask plaintext via multiplication is not recommended and DH value is used to derive the symmetric encryption key which is used to encrypt the plaintext in the semantically secure symmetric encryption. In order to prove the CCA security, CDH, DDH(Decisional Diffie Hellman) [9] and ICDH [10] assumptions are basically used. CCA security is a strong and very useful notion of security for PKE schemes [11–13].

In the random oracle model [14], hashed ElGamal is proved to be CCA secure (i.e., to be semantically secure against Chosen Ciphertext Attack) under the ICDH assumption and twin ElGamal is proved to be CCA secure under the CDH assumption [6, 7, 10]. Without random oracle model [3], Cramer-Shoup scheme is proved to be CCA secure under the DDH assumption. Table 1 shows the comparison between hashed ElGamal, Twin ElGamal and Cramer Shoup scheme.

**Table 1:** Comparison between hashed Elgamal and other CCA-secure Elgamal protocols in efficiency.

	Hashed ElGamal	Twin ElGamal	Cramer Shoup
Public Key	$g,u\leftarrow g^{x}$	$g,{u_{1}\leftarrow g}^{x_{1}},u_{2}\leftarrow g^{x_{2}}$	$g_{1},g_{2},{u_{1}\leftarrow g}_{1}^{x_{1}}g_{2}^{x_{2}},$ $u_{2}\leftarrow g_{1}^{y_{1}}g_{2}^{y_{2}},{u_{3}\leftarrow g}_{1}^{z}$
Private Key	*x*	*x*_1_, *x*_2_	$x_{1},x_{2},y_{1},y_{2},z$
Exponentiations in Encryption	${T\leftarrow g}^{r},u^{r}$	${T\leftarrow g}^{r},u_{1}^{r},u_{2}^{r}$	${T_{1}\leftarrow g}_{1}^{r},T_{2}\leftarrow g_{2}^{r},u_{3}^{r},$ $\alpha \leftarrow H(T_{1},T_{2},u_{3}^{r}m),$ ${\nu \leftarrow u}_{1}^{r}u_{2}^{r\alpha }$
Encryption	$c\leftarrow E_{s}(H(T,u^{r}),m)$	$c\leftarrow E_{s}(H(T,u_{1}^{r},u_{2}^{r}),m)$	$c\leftarrow u_{3}^{r}m$
Ciphertext	(*c*, *T*)	(*c*, *T*)	$\left(c,T_{1},T_{2},\nu \right)$
Exponentiations in Decryption	*T* ^*x*^	$T^{x_{1}},T^{x_{2}}$	$\alpha \leftarrow H(T_{1},T_{2},c)$ $T_{1}^{z},T_{1}^{x_{1}+y_{1}\alpha }T_{2}^{x_{2}+y_{2}\alpha }=\nu ?$
Decryption	$m\leftarrow D_{s}(H(T,T^{x}),c)$	$m\leftarrow D_{s}(H(T,T^{x_{1}},T^{x_{2}}),c)$	$m\leftarrow c/T_{1}^{z}$

Note. *g*, *g*_1_ and *g*_2_ denote the generators of multiplicative cyclic group. *H* denotes the hash function. *E*_*s*_ and *D*_*s*_ denote the symmetric encryption and decryption.

Note. ICDH assumption is noted as Strong DH assumption in [4, 7, 15–21]. However, in some papers [22–28], the name strong DH assumption also sometimes refers to a different assumption defined over bilinear maps. Hence, in order to avoid the conflict, Strong DH assumption is noted as ICDH assumption in [10] and this paper.

As shown in Table 1, among the above CCA secure protocols, hashed ElGamal is advantageous in the aspect of optimal ciphertext overhead(difference between ciphertext and plaintext) [7] and encryption/decryption efficiency. However, hashed ElGamal can be implemented only in ICDH group for the CCA security. At present, only bilinear group in which ICDH assumption is known to be equivalent to the CDH assumption [15] has been known as ICDH group.

However, bilinear group needs the special elliptic curve with more complex structure and so, CCA secure ElGamal system is most commonly implemented as twin ElGamal or Cramer-Shoup scheme in simple groups where CDH or DDH assumption holds. In other words, twin ElGamal or Cramer-Shoup scheme is commonly used instead of hashed ElGamal in practice.

To the best of our knowledge there are no results in the literature introducing the ICDH group with simple algebraic structure.

In this paper, we present a simple ICDH group and propose the CCA secure hashed ElGamal which has the possibility of fast decryption by parallel processing.

We highlight the following key results of our study:

- We prove that ICDH assumption holds in the integer group with composite modulus.
- In the integer group with composite modulus, we propose the hashed ElGamal and prove the CCA security.
- We modify the logical structure of hashed ElGamal to speed up decryption by parallel processing.

This paper is organized as follows. In Section2, we describe the important relevant preliminaries including PKE and reasonable assumptions. In Section 3, we analyze the CCA security of hashed ElGamal in *G* and propose a fast variant of hashed ElGamal in which decryption can be sped up by parallel processing. In Section 4, we show the some theoretical and experimental results of our implementation. In Section 5, we discuss the possibility of further reducing decryption time. Finally, we conclude with Section 6.

## 2. Preliminaries

A PKE scheme is a triple of algorithm (*K*, *E*, *D*) such that

- Key generation algorithm *K*: is a probabilistic algorithm that generates a pair of public and private keys (*pk*, *sk*).
- Encryption algorithm *E*: is a probabilistic algorithm that produces ciphertext *c*←*E*(*pk*, *m*) for given message *m* and public key *pk*.
- Decryption algorithm *D*: is a deterministic algorithm that outputs message *m*←*D*(*sk*, *c*) or special reject value ⊥ for given ciphertext *c* and private key *sk*.

For each pairs of key (*pk*, *sk*) generated by algorithm *K*, and for every message m,Pr[D(sk,E(pk,m))=m]=1.

The security of a PKE usually proved under a reasonable assumption. A typical assumption is the computational assumption which is described as the intractability of inverting problems such as factoring a composite number, computing the RSA problem, computing the DL(Discrete Logarithm) problem, and computing the CDH problem. In this case, an inverting problem is, given *y* and relation *f*, to find its solution, *x* satisfying *f*(*x*, *y*) = 1.

Another type of reasonable assumption is described as the intractability of the decision problem such as the DDH problem, which is usually used to prove the CPA(Chosen Plaintext Attack) security of PKE. A decision problem is, given (*x*, *y*) and *f*, to decide whether the pair (*x*, *y*) satisfies *f*(*x*, *y*) = 1 or not.

Let, $q,\frac{p-1}{2},\frac{q-1}{2}$ be prime numbers and *n* = *pq*. Let *G* be the multiplicative subgroup of $Z_{n}^{*}$ with generator *g* of order $\lambda =\frac{\left(p-1)(q-1\right)}{2}$. Then, above problems are described as follows.

Factoring problem: given a composite integer *n* = *pq* where the *p* and *q* are the safe primes, find *p* and *q*.

RSA problem: given *y*, find an integer *x* such that $x^{e}\equiv y(mod\;n)$.

DL problem: given a pair $\left(g,g^{x}|x\in Z_{n}\right)$, find the *x*.

CDH problem: given a triple $\left(g,g^{x},g^{y}|x,y\in Z_{n}\right)$, find the element *Y* = *g*^*xy*^.

DDH problem: given a quadruple of elements $\left(g,g^{x},g^{y},g^{z}|x,y,z\in Z_{n}\right)$, decide whether *z* = *xy mod λ* or not.

In order to prove the CCA security, strong type of reasonable assumption like Gap DH(Gap Diffie Hellman) assumption [17, 29–35] or ICDH assumption is usually used, which describes the intractability to solve an inverting problem with the access to the oracle of a related decision problem. A typical problem is, given *y* and *f*, to find *x* such that *f*(*x*, *y*) = 1, with the access to the oracle of, given question (*x*_1_, *y*_1_), answering whether *f*(*x*_1_, *y*_1_) = 1 or not. Gap DH and ICDH assumption in *G* are described in following section.

## 3. System model

We considered the integer group with composite modulus which is known as DDH group and proved that ICDH assumption holds in this group. In other words, we have proved that breaking generalized ICDH assumption modulo a composite leads to breaking RSA assumption [36] and, on the basis of this, proposed CCA secure hashed ElGamal in *G*.

In group *G*, CDH and DDH have been believed to be intractable [9, 37, 38]. Let (*n*, *e*) be the RSA public key and *d* be the RSA private key such that *ed*≡1 *mod λ*. Assume that an adversary can obtain the generator *g* of group *G* and *g*^*d*^(∈*G*)(In RSA, this is possible by randomly selecting generator *u* and setting *g* = *u*^*e*^*mod n*. In this case, *g* is also a generator and *u* = *g*^*d*^*mod n* is satisfied). And assume that *r* be the element of *G*.

Then, *r* = *g*^*x*^ is satisfied for some *x*(∈*Z*_*n*_) and if CDH assumption is broken in *G*, the adversary can obtain *r*^*d*^ (= *g*^*xd*^) from *r* (= *g*^*x*^) and *g*^*d*^.

From the fact above, it can be seen that breaking CDH assumption in group *G* gives the possibility to break the RSA assumption.

Note. Of course, CDH assumption has been already known to be intractable in *G* [37, 38]. In this paper, we reconsidered it in correlation with RSA assumption.

Similarly, we proved that ICDH assumption holds in *G* under the RSA assumption as follows.

In the ICDH problem, access to “DH-decision oracle” is added to CDH problem. Assume that CDH assumption is not broken, but ICDH assumption is broken in *G*. Then, the adversary can briefly break RSA assumption by using public key *e* as follows.

In RSA, the adversary can briefly test whether any triple $\left(u=g^{d},\hat{v},\hat{w}\right)$ he likes is a DH-triple (i.e., ${\hat{v}}^{d}=\hat{w}$ for the triple $\left(g^{d},\hat{v},\hat{w}\right)$) by using the given public key *e* (i.e., by checking that ${\hat{w}}^{e}=\hat{v}$), without knowledge of any secret key material and so, he never needs to issue queries to the challenger. In other words, the adversary can access the “DH-decision oracle” that recognizes DH-triples of the form (*g*^*d*^,∙,∙) offline on his own.

Note that in hashed ElGamal, the adversary has to access the “DH-decision oracle” online (more precisely, the adversary has to issue the decryption queries to the challenger in the “DH-decision oracle”) [7, 10].

Consequently, the adversary can obtain *r*^*d*^ (= *g*^*xd*^) from *r* (= *g*^*x*^) and *g*^*d*^ by using his own “DH-decision oracle” and so, it can be seen that breaking ICDH assumption in group *G* also gives the possibility to break the RSA assumption. See the proof of Theorem2 for more details.

When modulus *n* is large enough (2048bit), RSA assumption is not broken. Hence, in group *G*, ICDH assumption holds and hashed ElGamal is CCA secure for the large modulus.

Next, we modified the hashed ElGamal a little to speed up decryption by parallel processing. We converted the large private key to the group of small private keys and modified the encryption process so that small private keys are used in decryption. In this case, it is possible to speed up decryption by parallel processing. The results of modification are encouraging and show that hashed ElGamal can be still practical even when the big modulus number is used to resist the quantum computing.

### 3.1 CCA secure hashed ElGamal in integer group with composite modulus

The most important security guarantee needed for PKE is semantic security. Semantic security is classified into CPA security(Semantic security against chosen-plaintext attacks) and CCA security(Semantic security against adaptive chosen-ciphertext attacks) which are described as follows.

Algorithm 3.1: Chosen plaintext attack game, played between a challenger and adversary *A*.

**Step1**. The challenger generates a public key/private key pair (*pk*, *sk*), and sends the public key *pk* to *A*.

**Step2**. *A* makes one challenge query, which is a pair of messages (*m*_0_, *m*_1_) and sends them to the challenger.

**Step3**. The challenger chooses *b*∈{0, 1} at random, encrypts *m*_*b*_, and sends the ciphertext $c^{*}\leftarrow E(pk,m_{b})$ to *A*.

**Step4**. *A* outputs $\hat{b}\in \{0,1\}$.

The advantage of adversary is defined as ${Adv}_{cpa}=|Pr[\hat{b}=b]-1/2|.$ The scheme PKE is secure against chosen plaintext attack if for all efficient adversaries *A*, the advantage *Adv*_*cpa*_ is negligible.

Algorithm 3.2: Chosen ciphertext attack game, played between a challenger and adversary *A*.

**Step1**. The challenger generates a public key/private key pair (*pk*, *sk*), and sends the public key *pk* to *A*.

**Step2**. *A* makes a number of decryption queries to the challenger; each query is a ciphertext *c*; the challenger decrypts *c*, and sends the result *m*←*D*(*sk*, *c*) to *A*.

**Step3**. *A* makes one challenge query, which is a pair of messages (*m*_0_, *m*_1_) and sends them to the challenger.

**Step4**. The challenger chooses *b*∈{0, 1} at random, encrypts *m*_*b*_, and sends the ciphertext c*←*E*(*pk*, *m*_*b*_) to *A*.

**Step5**. *A* makes more decryption queries, just as in Step 2, but with the restriction that *c*≠c*;

**Step6**. *A* outputs $\hat{b}\in \{0,1\}$.

The advantage of adversary is defined as ${Adv}_{cca}=|Pr[\hat{b}=b]-1/2|$. The scheme PKE is secure against chosen ciphertext attack if for all efficient adversaries *A*, the advantage *Adv*_*cca*_ is negligible.

Note. A function *ε*(*k*) is said to be negligible if for every ***i***>0 there exists *k*_0_ satisfying $(k)<\frac{1}{k^{i}}$ for all *k*>*k*_0_.

If the security of PKE is proved in the random oracle model, hash functions are replaced by random oracle queries, and both challenger and adversary are allowed to access the random oracle in the above attack games.

In group *G*, we propose a CCA secure ElGamal whose ciphertext overhead consists of only one group element as follows.

Algorithm 3.3: Key generation for hashed ElGamal in *G*.

Each user creates the public key and the corresponding private key.

**Step1.** Select a multiplicative cyclic group *G* of order $\lambda (=\frac{\left(p-1)(q-1\right)}{2})$, with generator *g* where *p*, *q*, $\frac{p-1}{2}$ and $\frac{q-1}{2}$ are large primes.

In this case, *G* becomes a subgroup of $Z_{n(=pq)}^{*}$. This can be described in detail as follows.

**Step1.1.** Select the large primes *p*, *q*, *p*′ and *q*′ such that *p* = 2*p*′+1 and *q* = 2*q*′+1 and calculate n←pq,λ←2p′q′(=lcm(p−1,q−1)) and *q*_*inv*_ = *q*^−1^*mod p*.

**Step1.2.** Select the generator *g*_*p*_ of $Z_{p}^{*}$ and generator *g*_*q*_ of $Z_{q}^{*}$ and calculate $g(\in Z_{n}^{*})$ that satisfies *g*_*p*_ = *g mod p* and *g*_*q*_ = *g mod q* as follows.


$$g\leftarrow ((g_{p}-g_{q})q_{inv}\;mod\;p)q+g_{q}$$

In this case, *g* becomes a generator of *G*.

**Step2.** Select a random integer x(1≤x<λ,gcd(x,λ)=1) and compute the group element *u*←*g*^*x*^.

This can be described in detail as follows.

**Step2.1.** Select random integers $x_{p}(1<x_{p}<p-1)$ and $x_{q}(1<x_{q}<q-1)$ such that $\gcd\left(x_{p},p-1\right)=1$ and $\gcd\left(x_{q},q-1\right)=1$. In this case, $x_{p}\equiv x_{q}\;mod\;2$ is satisfied.

**Step2.2.** Calculate $u_{p}\leftarrow g_{p}^{x_{p}}mod\;p,u_{q}\leftarrow g_{q}^{x_{q}}mod\;q$ and

$$u\leftarrow ((u_{p}-u_{q})q_{inv}\;mod\;p)q+u_{q}.$$

In this case, $u=g^{x}mod\;n,x_{p}=x\;mod(p-1)$ and $x_{q}=x\;mod(q-1)$ are satisfied.

**Step3.** Public key is (*g*, *u*, *n*) and private key is *x*.

This can be described in detail as follows.

**Step3.1.** Public key is (*g*, *u*, *n*) and private key is (*x*, *x*_*p*_, *x*_*q*_, *p*, *q*).

Encryption and decryption use the symmetric encryption (*E*_*s*_, *D*_*s*_) defined over (*K*_*s*_, *M*_*s*_, *C*_*s*_) and hash function *H*(*G*^2^→*K*_*s*_).

Algorithm 3.4: Encryption for hashed ElGamal in *G*.

User encrypts a message *m*∈*M*_*s*_, where *M*_*s*_ is a message space of (*E*_*s*_, *D*_*s*_).

**Step1.** Obtain authentic public key (*g*, *u*, *n*).

**Step2.** Select a random integer *y*(1<*y*<*n*) and compute group elements *v*←*g*^*y*^, *w*←*u*^*y*^ and hash value *k*_*s*_←*H*(*v*, *w*).

**Step3.** Encrypt the message *m* by using symmetric encryption *E*_*s*_ and key *k*_*s*_.


$$c\leftarrow E_{s}(k_{s},m)$$

**Step4.** Send the cipher text (*v*∈*G*, *c*∈*C*_*s*_). *C*_*s*_ is a cipher text space of (*E*_*s*_, *D*_*s*_).

Algorithm 3.5: Decryption for hashed ElGamal in *G*.

User recovers message *m* from (*v*, *c*).

**Step1.** Compute the group element *w*←*v*^*x*^ and hash value *k*_*s*_←*H*(*v*,*w*).

Calculation of *w* can be done fast by using CRT(Chinese Remainder Theorem) exponents *x*_*p*_ and *x*_*q*_ as in CRT-RSA [39].

**Step1.1.** Calculate $v_{p}\leftarrow v\;mod\;p,v_{q}\leftarrow v\;mod\;q$ and $q_{inv}=q^{-1}mod\;p$.

**Step1.2.** Calculate

$$w_{p}\leftarrow v_{p}^{x_{p}}mod\;p$$

and

$$w_{q}\leftarrow v_{q}^{x_{q}}mod\;q.$$

**Step1.3.** Calculate *w* as follows.


$$w\leftarrow ((w_{p}-w_{q})q_{inv}\;mod\;p)q+w_{q}$$

**Step1.4.** Calculate *k*_*s*_←*H*(*v*, *w*).

**Step2.** Recover the message *m* by using symmetric decryption *D*_*s*_ and key *k*_*s*_.


$$m\leftarrow D_{s}(k_{s},c)$$

Because CDH and DDH assumptions are satisfied in *G* [9], following Theorem1 can be obtained referring to [10].

**Theorem 1. If** $H:{Z_{n}^{*}}^{2}\to K_{s}$ **is modeled as a random oracle and symmetric encryption (*E*_*s*_, *D*_*s*_) is CPA secure (i.e., is semantically secure against Chosen Plaintext Attack), then hashed ElGamal in *G* is CPA secure.**

***Proof***. Assume that there exists an IND(Indistinguishability)-CPA adversary *A* which makes at most *Q* queries to the random oracle and has advantage *ε*_*EG*_ in hashed ElGamal. Then, we present CDH adversary *B*_*cdh*_ which has advantage *ε*_*cdh*_ in group *G* and IND-CPA adversary *B*s which has advantage *ε*_*s*_ in symmetric encryption (*E*_*s*_, *D*_*s*_) such that

$$\varepsilon_{EG}\leq Q∙\varepsilon_{cdh}+\varepsilon_{s}$$ (1)

We define Game1 as a modified version of Game0, which is the actual attack game to hashed ElGamal in *G*. In each game, *b* denotes the random bit chosen by the challenger, while $\hat{b}$ denotes the bit output by *A*. For *j* = 0, 1, we define *W*_*j*_ to be the event that $\hat{b}=b$ in Game *j*.

From the assumption,

$$|Pr[W_{0}]-1/2|=\varepsilon_{EG}.$$ (2)

**Game 0.** Challenger selects *x*, *y* randomly so that $x,\;y\in Z_{\lambda },\gcd\left(x,\lambda \right)=1$ and calculates $u=g^{x},v=g^{y},w=g^{xy}$.

The random oracle is implemented by using an array $Map:{Z_{n}^{*}}^{2}\to K$. Challenger selects *k*∈*K* randomly and sets *Map*[*v*, *w*] = *k*. And challenger sends the public key *u* to adversary *A*. Then, adversary *A* outputs a pair of messages (*m*_0_, *m*_1_) and challenger produces the ciphertext (*v*, *c* = *E*_*s*_(*k*, *m*_*b*_)) by flipping a coin *b*.

- When random oracle is queried at $(\hat{v},\hat{w})\in {Z_{n}^{*}}^{2}$, challenger acts as follows.

If $Map[\hat{v},\hat{w}]=\emptyset$ then select *k*∈*K* randomly and set $Map[\hat{v},\hat{w}]=k$.

The answer corresponding to random oracle query at $\left(\hat{v},\hat{w}\right)$ is $Map[\hat{v},\hat{w}]$.

**Game 1.** We modify Game0 by setting *Map*[*v*, *w*] = ∅ instead of *Map*[*v*, *w*] = *k*.

Let *Z* be the event that the adversary queries the random oracle at (*v*, *w*) in Game 1.

Then

$$|Pr[W_{1}]-Pr[W_{0}]|\leq Pr[Z].$$ (3)

If event *Z* happens, then one of the adversary’s random oracle queries is (*v*, *w*), where *w* = *v*^*x*^.

Also, challenger uses *x* and *y* only to compute *u* and *v* in Game1.

Hence, we can use adversary *A* to build adversary *B*_*cdh*_ to break the CDH assumption. *B*_*cdh*_ chooses one of the *A*’s random oracle queries $\left(\hat{v},\hat{w}\right)$ at random, and the probability that such $\hat{w}=w$ will be chosen from random selection is at least *Pr*[*Z*]/*Q*. In other words,

$$\varepsilon_{cdh}\geq Pr[Z]/Q.$$ (4)

Meanwhile, in Game 1, the key *k* is used only to encrypt the challenge plaintext.

Hence, we can also use adversary *A* to build IND-CPA adversary *B*_*s*_ in symmetric encryption (*E*_*s*_, *D*_*s*_).

From the definition of IND-CPA adversary,

$$|Pr[W_{1}]-1/2|=\varepsilon_{s}$$ (5)

By combining (2), (3), (4) and (5), we can obtain (1). **(end of proof)**

Theorem1 shows only the CPA security of hashed ElGamal in *G*. For the CCA security, a stronger assumption is needed.

Assume that the adversary selects arbitrary elements $\hat{v}(\in Z_{n}^{*})$ and $\hat{w}(\in Z_{n}^{*})$, and computes ${\hat{k}}_{s}=H(\hat{v},\hat{w})$ and $\hat{c}=E_{s}({\hat{k}}_{s},\hat{m})$ for some arbitrary message $\hat{m}(\in M_{s})$. Further, assume the adversary gives the ciphertext $\left(\hat{v},\hat{c}\right)$ to a “decryption oracle” and obtains the decryption $m=D_{s}(H(\hat{v},{\hat{v}}^{x}),\hat{c})$. Now, it is very likely that $m=\hat{m}$ if and only if $\hat{w}={\hat{v}}^{x}$. See [7] and [10] for more details.

Note. Decryption algorithm does not verify that $\hat{v}\in G$ (Of course, such a verification can be easily done, but it requires additional calculation. Furthermore, it could present a more attractive target for the adversary because it gives an oracle to check whether or not $\hat{v}\in G$? for an arbitrary element $\hat{v}\in Z_{n}^{*}$) for given ciphertext $\left(\hat{v},c\right)$ (See Algorithm3.5) and so, $\hat{v}\in Z_{n}^{*}$ and $\hat{w}\in Z_{n}^{*}$ can be used instead of $\hat{v}\in G$ and $\hat{w}\in G$, respectively, in the CCA scenario (more precisely, in the definition of DH-triple $\left(u,\hat{v},\hat{w}\right)$).

For $U(=g^{x})\in G,V\in Z_{n}^{*}$, define the predicate *dh*(*U*, *V*)≔*V*^*x*^ and for $U\in G,\hat{V},\hat{W}\in Z_{n}^{*}$, define the predicate $dhp(U,\hat{V},\hat{W})≔(dh(U,\hat{V})=\hat{W}?)$. (These are little different from the definition of [7, Section1.1] and [10, Section11.4] because $\hat{V},\hat{W}\in Z_{n}^{*}$ are used instead of $\hat{V},\hat{W}\in G$. As mentioned above, factorization of *n* is unknown and so, adversary cannot distinguish between *G* and $Z_{n}^{*}$.) Then, in the CCA scenario, the adversary can use the decryption oracle to answer questions (i.e., $\hat{w}={\hat{v}}^{x}$?) of the form $dhp(u=g^{x},\hat{v},\hat{w})$ for elements $\hat{v}(\in Z_{n}^{*})$ and $\hat{w}(\in Z_{n}^{*})$ of the adversary’s choosing.

The adversary cannot efficiently answer such questions on his own(if he can, DDH assumption is broken in *G*), and so the decryption oracle is leaking some information about that secret key *x* which could potentially be used to break the encryption scheme.

From the facts above, ICDH assumption which is used in the CCA security of hashed ElGamal over *G* can be defined as follows.

**ICDH assumption:** It is difficult to compute *dh*(*U*, *V*), given random *U*∈*G* and *V*∈*G*, along with access to decision oracle for the predicate *dhp*(*U*,∙,∙), which on input $\left(\hat{V}\in Z_{n}^{*},\hat{W}\in Z_{n}^{*}\right)$, returns $dhp(U,\hat{V},\hat{W})$.

Note. Gap DH assumption where an adversary gets access to a full DH decision oracle for the predicate *dhp*(∙,∙,∙), which on input $\left(\hat{U},\hat{V},\hat{W}\right)$, returns $dhp(\hat{U},\hat{V},\hat{W})$ is different (and stronger) than ICDH assumption where an adversary gets access to a restricted DH decision oracle for the predicate *dhp*(*U*,∙,∙), which on input $\left(\hat{V},\hat{W}\right)$, returns $dhp(U,\hat{V},\hat{W})$. In other words, ICDH assumption (where the first element of the triplets submitted to the DH decision oracle is fixed) is implied by the Gap DH assumption (where the first element can be freely chosen) [7, 17, 40, 41].

Following Theorem2 shows that if ICDH assumption is broken in *G*, then it is possible to break RSA assumption.

Theorem 2: Assume ICDH assumption is (*t*, *q*_*dh*_, *ε*)-broken in group *G*, where *q*_*dh*_ is the number of queries to “DH-decision oracle” and *ε* is the probability to break the assumption in time *t*. Then, RSA assumption is (*t*, *q*_*dh*_, *ε*/8)-broken when safe primes are used.

***Proof***. Let *B* be an attacker which (*t*, *q*_*dh*_, *ε*)-breaks ICDH assumption in group *G*. We present an adversary *A* which (*t*, *q*_*dh*_, *ε*/8)-breaks RSA assumption when modulus *n* is the product of two safe primes. Let *e* be the public exponent and *d* be the private exponent. Adversary *A* is given as input (*n*, *e*, *r*) where *r* was chosen at random from $Z_{n}^{*}$ and is trying to find *r*^*d*^ *mod n*.

In RSA, anyone can obtain the pair of elements (*h*, *h*^*d*^), where *h* is an element of $Z_{n}^{*}$, by selecting arbitrary element $u\in Z_{n}^{*}$ and setting *h* = *u*^*e*^*mod n*(i.e., *u* = *h*^*d*^*mod n*). Besides, anyone can obtain the arbitrary element $v\in Z_{n}^{*}$ by multiplying *e*^*th*^ power of arbitrary element $s\in Z_{n}^{*}$ and *r* (i.e., *v* = *s*^*e*^*r mod n*).

Assume that *h* is a generator of *G* and *v* is an element of *G*(i.e., *v* = *h*^*a*^).

Then, the ICDH attacker *B* can obtain *v*^*d*^ = *h*^*ad*^ from elements *u* = *h*^*d*^ and *v* = *h*^*a*^ with success probability *ε* and running time *t*, making *q*_*dh*_ queries to “DH-decision oracle” that recognizes DH-triples of form $\left(h^{d}\in G,∙\in Z_{n}^{*},∙\in Z_{n}^{*}\right)$.

In this case, “DH-decision oracle” is different from the one of hashed ElGamal.

First, in order to determine whether or not any triple $\left({u=h}^{d}\in G,\hat{v}\in Z_{n}^{*},\hat{w}\in Z_{n}^{*}\right)$ is DH-triple(i.e., ${\hat{v}}^{d}=\hat{w}$?), the ICDH attacker *B* checks that ${\hat{w}}^{e}=\hat{v}$ using RSA public exponent *e* on his own without making queries to the challenger, because modular inverse of private key (i.e., *e* = *d*^−1^*mod λ*) is published in RSA, unlike hashed ElGamal. In other words, “DH-decision oracle” can be done off line(This creates more favorable conditions to *B* than in hashed ElGamal’s DH-decision oracle) by *B* and so, *A* need not simulate “DH-decision oracle” to answer *B*’s query.

Second, the computational cost per iteration of “DH-decision oracle” query is comparable to hashed ElGamal.

In RSA, small public exponents are commonly used (i.e., RSA assumption still holds for small public exponents such as 3 and 65537) and so, for given $\left(u,\hat{v},\hat{w}\right)$, calculation of $\tilde{v}={\hat{w}}^{e}$ for the test ($\tilde{v}=\hat{v}?$) is much faster(This also creates favorable conditions to *B*) than the calculation of “DH-decision oracle” of hashed ElGamal in *G* (i.e., calculation of $\hat{k}=H(\hat{v},\hat{w}),\hat{c}=E_{s}(\hat{k},\hat{m}),\tilde{w}={\hat{v}}^{x},k=H(\hat{v},\tilde{w})$ and $m^{*}=D_{s}(k,\hat{c})$ for the test ($\hat{m}=m^{*}?$)) because *log*_*n*_*x*≈1. Even though full sized public exponent *e*(*log*_*n*_*e*≈1) is used [42] in RSA, computation of ${\hat{w}}^{e}$ is comparable to the computation of ${\hat{v}}^{x}$ of decryption oracle in hashed ElGamal.

Of course, the generator and element of *G* are unknown to *B*. Hence, adversary *A* must select *h* (= *u*^*e*^) and *v* (= *s*^*e*^*r mod n*) as a generator and an element of *G*, respectively, and run the ICDH attacker *B* on input (*u*(= *h*^*d*^),*v*) in order to get *v*^*d*^.

Meanwhile, many elements of $Z_{n}^{*}$ can become the generator or element of *G*. Hence, when adversary *A* selects *h* and *v* as random elements of $Z_{n}^{*}$ (this is accomplished by anyone in RSA as mentioned above), *h* becomes a generator and *v* becomes an element of *G* with high probability.

Let $p\prime =\frac{p-1}{2}$ and $q\prime =\frac{q-1}{2}$. From Algorithm3.3, the order of *G* is *λ* = 2*p*′*q*′ and so, the probability that random element *v*∈*Z*_*n*_ is included in *G* is as follows.


$$\mathrm{Pr}\left[Element(v,G)=1|v\in Z_{n}\right]=\frac{\lambda }{n}=\frac{2p^{\prime }q^{\prime }}{\left(2p\prime +1)(2q\prime +1\right)}=\frac{2p^{\prime }q^{\prime }}{4p\prime q\prime +2(p\prime +q\prime )+1}\approx \frac{{2p}^{\prime }q^{\prime }}{4p^{\prime }q^{\prime }}=\frac{1}{2}$$ (6)

The group of order *λ* has *ϕ*(*λ*) generators, where *ϕ* is Euler phi function. Hence, the probability that random element *h*∈*Z*_*n*_ becomes a generator of *G* is as follows.


$$\mathrm{Pr}\left[Genertor(h,G)=1|h\in Z_{n}\right]=\frac{\phi (\lambda )}{n}=\frac{\left(p\prime -1)(q\prime -1\right)}{\left(2p\prime +1)(2q\prime +1\right)}=\frac{p\prime q\prime -(p\prime +q\prime )+1}{4p\prime q\prime +2(p\prime +q\prime )+1}\approx \frac{p^{\prime }q^{\prime }}{4p^{\prime }q^{\prime }}=\frac{1}{4}$$ (7)

From Eqs (6) and (7), the probability that *h* is a generator of *G* and *v* is included in *G* for arbitrarily selected *h* and *v* is as follows.


$$\mathrm{Pr}\left[Genertor(h,G)=1,Element(v,G)=1h\in Z_{n},v\in Z_{n}\right]\approx \frac{1}{4}∙\frac{1}{2}=\frac{1}{8}$$ (8)

Hence, with probability at least 1/8, *A* can select *h* and *v* as a generator and element of *G*, respectively, and give *B* the challenge instance (*u* = *h*^*d*^, *v* = *h*^*a*^). If and when *B* outputs *v*^*d*^*mod n*, *A* outputs *r*^*d*^*mod n* = *v*^*d*^*s*^−1^*mod n*.

From all facts above, it can be seen that if ICDH assumption is (*t*, *q*_*dh*_, *ε*)-broken in *G*, then it is possible to (*t*, *q*_*dh*_, *ε*/8)-break RSA assumption.**(end of proof)**

Even though safe primes *p* and *q* are used, RSA assumption have been believed not to be broken(regardless of whether public exponent *e* is small or large) and so, ICDH assumption holds in *G* from Theorem2.

From the above fact, referring to [10], following Theorem3 can be obtained.

**Theorem 3. If** $H:{Z_{n}^{*}}^{2}\to K_{s}$ is modeled as a random oracle and symmetric encryption (*E*_*s*_, *D*_*s*_) is CCA secure(i.e., is semantically secure against Chosen Ciphertext Attack), then hashed ElGamal in *G* **is CCA secure.**

***Proof***. Assume that there exists an IND-CCA adversary *A* which has advantage *ε*_*EG*_ in hashed ElGamal. Then, we present ICDH adversary *B*_*icdh*_ which has advantage *ε*_*icdh*_ in group *G* and IND-CCA adversary *B*s which has advantage *ε*_*S*_ in symmetric encryption (*E*_*s*_, *D*_*s*_) such that

$$\varepsilon_{EG}\leq \varepsilon_{icdh}+\varepsilon_{s}.$$ (9)

We define Game1 as a modified version of Game0, which is the actual attack game to hashed ElGamal in *G*. In each game, *b* denotes the random bit chosen by the challenger, while $\hat{b}$ denotes the bit output by *A*. For *j* = 0, 1, we define *W*_*j*_ to be the event that $\hat{b}=b$ in Game *j*.

From the assumption,

$$|Pr[W_{0}]-1/2|=\varepsilon_{EG}.$$ (10)

**Game 0.** Challenger selects *x*, *y* randomly so that $x,y\in Z_{\lambda },\gcd\left(x,\lambda \right)=1$ and calculates $u=g^{x},v=g^{y},w=g^{xy}$. The random oracle is implemented by using array $Map:{Z_{n}^{*}}^{2}\to K,Map’:Z_{n}^{*}\to K$ and $Sol:Z_{n}^{*}\to Z_{n}^{*}$. Challenger selects *k*∈*K* randomly and sets Map[v,w]=Map′[v]=k. And challenger sends the public key *u* to adversary *A*. Then, adversary *A* outputs a pair of messages (*m*_0_, *m*_1_) and challenger produces the ciphertext (*v*, *c* = *E*_*s*_(*k*, *m*_*b*_)) by flipping a coin *b*.

- When decryption oracle is queried at $(\hat{v},\hat{c})\in Z_{n}^{*}\times C$ where $\left(\hat{v},\hat{c})\neq (v,c\right)$, challenger acts as follows.

If $\hat{v}=v$ then $\hat{m}=D_{s}(k,\hat{c})$.

Else

If $Map\prime [\hat{v}]=\emptyset$ then select *k*∈*K* randomly and set $Map^{\prime }[\hat{v}]=k$.


$$\hat{m}=D_{s}(Map^{\prime }[\hat{v}],\hat{c}).$$

$\hat{m}$ is the answer corresponding to decryption oracle query at $\left(\hat{v},\hat{c}\right)$.

- When random oracle is queried at $(\hat{v},\hat{w})\in {Z_{n}^{*}}^{2}$, challenger acts as follows.

If $Map[\hat{v},\hat{w}]=\emptyset$ then

If $dhp(u,\hat{v},\hat{w})$ then

If $Map\prime [\hat{v}]=\emptyset$ then select *k*∈*K* randomly and set $Map^{\prime }[\hat{v}]=k.$

$Map[\hat{v},\hat{w}]=Map^{\prime }[\hat{v}],Sol[\hat{v}]=\hat{w}$.

Else

Select *k*∈*K* randomly and set $Map[\hat{v},\hat{w}]=k$.

The answer corresponding to random oracle query at $\left(\hat{v},\hat{w}\right)$ is $Map[\hat{v},\hat{w}]$.

**Game 1.** We modify Game0 by setting Map[v,w]=Map′[v]=∅ instead of Map[v,w]=Map′[v]=k.

Let *Z* be the event that the adversary queries the random oracle at (*v*, *w*) in Game 1.

Then

$$|Pr[W_{1}]-Pr[W_{0}]|\leq Pr[Z].$$ (11)

If event *Z* happens, then *Sol*[*v*] = *w*. Moreover, in Game1, challenger uses *x* only to compute *u* and to evaluate *dhp* function. Meanwhile, from the assumption, *B*_*icdh*_ can use the DH-decision oracle(i.e., can evaluate *dhp* function without *x*).

Hence, we can use adversary *A* to build an adversary *B*_*icdh*_ to break the ICDH assumption and *B*_*icdh*_ outputs *w* = *Sol*[*v*] with probability *Pr*[*Z*].

In other words,

$$\varepsilon_{icdh}=Pr[Z].$$ (12)

Meanwhile, in Game1, the key *k* is used only to encrypt the challenge plaintext, and to process decryption queries of the form $\left(v,\hat{c}\right)$, where $\hat{c}\neq c$.

Hence, we can also use adversary *A* to build IND-CCA adversary *B*_*s*_ in symmetric encryption (*E*_*s*_, *D*_*s*_). From the definition of IND-CCA adversary,

$$|Pr[W_{1}]-1/2|=\varepsilon_{s}.$$ (13)

By combining (10), (11), (12) and (13), we can obtain (9). **(end of proof)**

Composite number is used as modulus number and so, CRT can be used to speed up decryption of hashed ElGamal in *G*. However, in decryption, this scheme is still not fast because big prime numbers(1024bit and 7680bit prime numbers are needed to be secure from the current attacks and quantum computing attacks, respectively.) are used. To increase the decryption speed by parallel processing, we modified the logical structure of hashed ElGamal as follows.

### 3.2 Parallel scheme

Let *T*_*d*_ denotes the decryption time of a single ciphertext block which has the bitlength of modulus and *N* denotes the number of processors. Then, it is trivial that *N* ciphertext blocks can be decrypted by *N* processors in time *T*_*d*_ using parallel processing. However, this does not mean that a single ciphertext block can be decrypted in time *T*_*d*_/*N*. In other words, no message is recovered in time *T*_*d*_/*N* even by parallel processing in hashed ElGamal. In order to decrypt a single ciphertext block in time *T*_*d*_/*N* by parallel processing, we modify the logical structure of hashed ElGamal as follows.

Key generation is same as the hashed ElGamal in *G* and so, we describe only the encryption and decryption algorithms.

Encryption and decryption use the CCA secure symmetric encryption (*E*_*s*_, *D*_*s*_) defined over (*K*_*s*_, *M*_*s*_, *C*_*s*_) and hash function *H*(*G*^2^→*K*_*s*_).

Algorithm 3.6: Encryption for parallel scheme.

User encrypts a message *m*∈*M*_*s*_, where *M*_*s*_ is a message space of (*E*_*s*_, *D*_*s*_).

**Step1.** Obtain authentic public key (*g*, *u*, *n*) and set *h*←2^⌈*r*/2⌉^ and *g*_1_←*g*^*h*^, where *n* is 2*r*-bit number.

**Step2.** Select a random integer *y*(1<*y*<*n*) and compute group elements $v\leftarrow g^{y},v_{1}\leftarrow g_{1}^{y},w\leftarrow u^{y}$ and hash value *k*_*s*_←*H*(*v*,*w*). In this case, $v_{1}=g^{hy}={g^{yh}=v}^{h}$.

**Step3.** Encrypt the message *m* by using symmetric encryption *E*_*s*_ and key *k*_*s*_.


$$c\leftarrow E_{s}(k_{s},m)$$

**Step4.** Send the cipher text $\left(v\in G,v_{1}\in G,c\in C_{s}\right)$. *C*_*s*_ is a cipher text space of (*E*_*s*_, *D*_*s*_).

Algorithm 3.7: Decryption for parallel scheme.

User recovers message *m* from (*v*, *v*_1_, *c*).

**Step1.** Compute the group element *w*←*v*^*x*^ and hash value *k*_*s*_←*H*(*v*, *w*).

Calculation of *w* can be done fast by using *r*-bit CRT exponents $x_{p}\leftarrow hx_{1p}+x_{0p}$ and $x_{q}\leftarrow hx_{1q}+x_{0q}$, where *h* = 2^⌈*r*/2⌉^ and $0<x_{0p},x_{1p},x_{0q},x_{1q}<h$.

**Step1.1.** Calculate $v_{p}\leftarrow v\;mod\;p,v_{q}\leftarrow v\;mod\;q,v_{1p}\leftarrow v_{1}\;mod\;p,v_{1q}\leftarrow v_{1}\;mod\;q$ and $q_{inv}=q^{-1}mod\;p$.

**Step1.2.** Calculate

$$w_{p}\leftarrow v_{1p}^{x_{1p}}v_{p}^{x_{0p}}mod\;p(=v_{p}^{x_{p}}mod\;p)$$

and

$$w_{q}\leftarrow v_{1q}^{x_{1q}}v_{q}^{x_{0q}}mod\;q(=v_{q}^{x_{q}}mod\;q).$$

**Step1.3.** Calculate *w* as follows.


$$w\leftarrow ((w_{p}-w_{q})q_{inv}mod\;p)q+w_{q}$$

**Step1.4.** Calculate *k*_*s*_←*H*(*v*,*w*).

**Step2.** Recover the message *m* by using symmetric decryption *D*_*s*_ and key *k*_*s*_.


$$m\leftarrow D_{s}(k_{s},c)$$

In the Step1.2 of Algorithm3.7, $v_{1p}^{x_{1p}}\;mod\;p,v_{p}^{x_{0p}}\;mod\;p,v_{1q}^{x_{1q}}mod\;q$ and $v_{q}^{x_{0q}}mod\;q$ can be calculated in parallel and so, it seems that private key length is reduced to 1/2 in hashed ElGamal. (Of course, without parallel processing, the calculation of $v_{1p}^{x_{1p}}v_{p}^{x_{0p}}\;mod\;p$ and $v_{1q}^{x_{1q}}v_{q}^{x_{0q}}\;mod\;q$ can be done fast by simultaneous multiple exponentiation algorithm [37]).

In security, parallel scheme is identical to hashed ElGamal in *G*.

In parallel scheme, *h* (= 2^⌈*r*/2⌉^) does not provide any information except for the bit size of private key, which has been known to be approximately equal to modulus number’s bitlength(i.e., 2*r*). In other words, some $\left(g^{hy}mod\;n={g^{yh}mod\;n=v}^{h}mod\;n\right)$ of calculation needed in decryption (*v*^*d*^*mod n*) has been only pre-calculated at the encryption stage.

This can be seen from the following Theorem4.

Theorem4. Assume that parallel scheme is (*t*, *ε*)-broken, where *ε* is the probability to break the encryption scheme in time *t*. Then, hashed ElGamal in *G* is also (*t*, *ε*)-broken.

***Proof*.** Assume that *B* is an adversary which (*t*, *ε*)-breaks the one-wayness of parallel scheme. Then, we present adversary *A* which (*t*, *ε*)-breaks the one-wayness of hashed ElGamal in *G*. Let (*g*, *u*, *n*) be the public key and *x* be the private key of hashed ElGamal. Adversary *A* is given as input (*g*, *u*, *n*, *c*, *v*) and is trying to find the plaintext *m*, where (*c*, *v*) is the ciphertext. In hashed ElGamal, anyone knows the bit size of modulus number(i.e., 2*r*) and can obtain *h* = 2^⌈*r*/2⌉^.

Hence, *A* can obtain *h* (= 2^⌈*r*/2⌉^) and give *B* the challenge instance (*g*, *u*, *n*, *c*, *v*, *v*^*h*^). From the assumption, *B* is given as input (*g*, *u*, *n*, *c*, *v*, *v*^*h*^) and outputs $m=D_{s}(H(v,v^{x}),c)$. If and when *B* outputs *m*, *A* outputs *m*.

In the same way above, we can present the IND-CPA(or IND-CCA) adversary *A* to hashed ElGamal in *G* from the IND-CPA(or IND-CCA) adversary *B* to parallel scheme.

From all facts above, it can be seen that if parallel scheme is (*t*, *ε*)-broken, then it is possible to (*t*, *ε*)-break hashed ElGamal in *G*. **(end of proof)**

Similarly, it is possible to reduce the decryption time by setting *h* = 2^⌈*r*/3⌉^. In this case, ciphertext $\left(g^{y},g^{hy},g^{h^{2}y},c\right)$ can be decrypted in parallel by using private key $x_{0p},x_{1p},x_{2p},x_{0q},x_{1q},x_{2q}$ instead of $x_{p}={\left(x_{2p}x_{1p}x_{0p}\right)}_{h}$ and $x_{q}={\left(x_{2q}x_{1q}x_{0q}\right)}_{h}$, where (*X*_2_*X*_1_*X*_0_)_*h*_ is the base *h* representation of *X*. In other words, $g^{yx_{p}}\;mod\;p=g^{yx_{0p}}g^{y{hx}_{1p}}g^{yh^{2}x_{0p}}\;mod\;p$ and $g^{yx_{q}}mod\;q=g^{yx_{0q}}g^{y{hx}_{1q}}g^{yh^{2}x_{0q}}\;mod\;q$

In such way, it is possible to propose the fast ElGamal variants which is *t*(*t* = 2,3,4,…) times faster than ordinary hashed ElGamal in *G*. Of course, in this case, there is message expansion by a factor of *t*. However, when considering the current network throughput and the fact that PKE is used only to establish a session key, drawback caused from the message expansion could be ignored compared to the benefit gained by speed-up.

Note. Unlike the parallel scheme, the scheme of [43] compromises the security of hashed ElGamal because CRT exponents *x*_*p*_ and *x*_*q*_ are reduced.

## 4. Performance analysis

Let *t* denotes the number of processors participating in parallel processing. When 2*r* bits modulus number is used, expected decryption speed-up factor *β* can be denoted as follows.


$$\beta =\frac{r\times 0.5+r\times 0.5+2\times r+2}{\lceil \frac{r}{t}\rceil \times 0.5+\lceil \frac{r}{t}\rceil \times 0.5+2\times \lceil \frac{r}{t}\rceil +2\times \lceil {log}_{2}t\rceil +2}=\frac{r\times 3+2}{\lceil \frac{r}{t}\rceil \times 3+2\times \lceil {log}_{2}t\rceil +2}$$ (14)

Note. Let *T*_*M*_ denotes the modular multiplication time of two *r*-bits numbers. Then, *t* numbers can be multiplied in time ⌈*log*_2_*t*⌉ *T*_*M*_ by parallel processing with *μ*(≥⌈*t*/2⌉) processors.

Table 2 shows the theoretical decryption time comparison of CRT-RSA, hashed ElGamal in *G* and parallel scheme.

**Table 2:** Theoretical decryption time comparison between parallel scheme and CRT-RSA (2048-bit modulus).

	Hashed ElGamal in *G* (KEM/DEM)	Parallel scheme (*t* = 2) (KEM/DEM)	Parallel scheme (*t* = 4) (KEM/DEM)	CRT-RSA (OAEP)
Decryption Exponent (CRT Exponent)	2048 bits (1024 bits)	2048 bits (1024 bits)	2048 bits (1024 bits)	2048 bits (1024 bits)
Number of Multiplication in Decryption (Modular Size)	2×1024×1.5+2 = 3074 (1024 bits)	2×(512×1.5+1)+2 = 1540 (1024 bits)	2×(256×1.5+2)+2 = 774 (1024 bits)	2×1024×1.5+2 = 3074 (1024 bits)
Unit time for decryption	1	0.5	0.252	1

As shown in Table 2, *β*≈*t* is usually satisfied when *r* is much larger than *t*. However, Hamming weight(which is the number of ones in binary representation) of *r*-bits number is not always actually *r*/2 and so, exact decryption speed-up factor can be denoted as

$$\bar{\beta }=\frac{V+W+2\times r+2}{\max\left\{V_{i}|1\leq i\leq t\right\}+\max\left\{W_{i}|1\leq i\leq t\right\}+2\times \lceil \frac{r}{t}\rceil +2\times \lceil {log}_{2}t\rceil +2}$$ (15),

where *V*, *W*, *V*_*i*_ and *W*_*i*_ denotes the Hamming weights of *x*_*p*_, *x*_*q*_, *x*_*ip*_ and *x*_*iq*_, respectively. In Eq (15), we used *max*{*V*_*i*_|1≤*i*≤*t*} (or *max*{*W*_*i*_|1≤*i*≤*t*}) instead of $\sum_{i=1}^{t}V_{i}/t$ (or $\sum_{i=1}^{t}W_{i}/t$) considering the delay associated with synchronizing parallel processes.

If *w*_*p*_ and *w*_*q*_ are calculated simultaneously by using parallel processing (In this case, 2*t* processors are needed), then

$$\hat{\beta }=\frac{\max\left\{V,W\right\}+r+2}{\max\left\{\max\left\{V_{i}|1\leq i\leq t\right\},\max\left\{W_{i}|1\leq i\leq t\right\}\right\}+\lceil \frac{r}{t}\rceil +\lceil {log}_{2}t\rceil +2}$$ (16)

**Fig 1:**
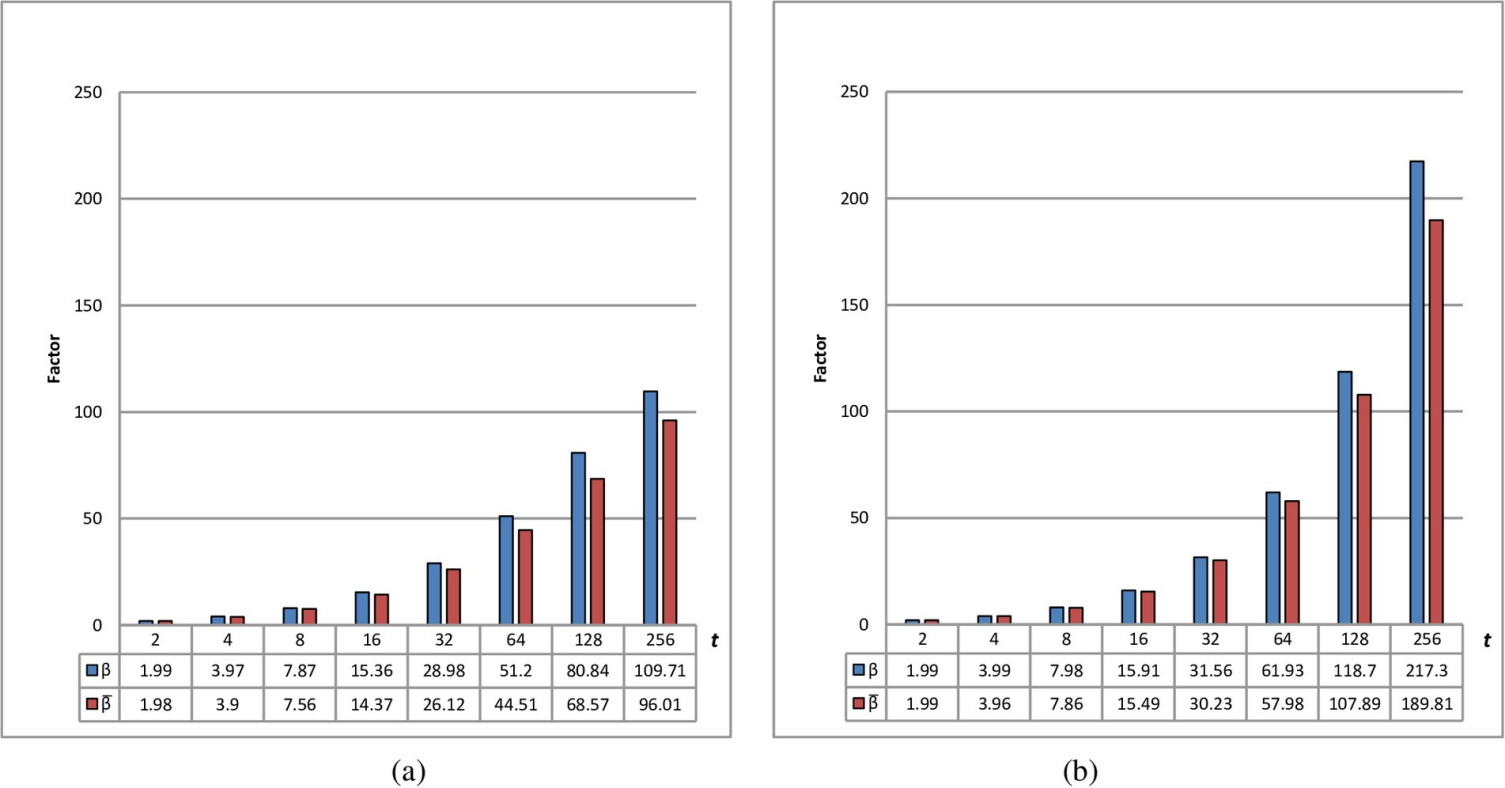
**Relation between *β* and $\bar{\beta }$ in different *t*.** (a): 2048-bit modulus. (b): 15768-bit modulus.

Fig 1 shows the relation between *β* and $\bar{\beta }$ in different *t* values. In order to obtain an average value of $\bar{\beta }$, we ran the key generation algorithm 1000 times, each of which included 100 different *x* values. When the common multicore CPUs of Intel or AMD are used in parallel processing, *t* is usually small(i.e., *t*<16). If many core GPUs of NVIDIA or multi-CPUs are used in parallel processing, then *t* is not small. However, it is not practical to set *t* too large(i.e., *t*>256) because of message expansion. As shown in Fig 1, $\bar{\beta }$ is slightly small than *β* because *V* and *W* are similar to *r*×0.5, but *max*{*V*_*i*_|1≤*i*≤*t*} and *max*{*W*_*i*_|1≤*i*≤*t*} are usually larger than $\lceil \frac{r}{t}\rceil \times 0.5$ (See S1 to S4 Tables). Meanwhile, the effectiveness (*β*/*t* or $\bar{\beta }/t$) decreases with increasing the number of processors and increases with increasing modulus number.

Consequently, our scheme gives the possibility to propose the fast public key cryptosystem which is approximately $\bar{\beta }(4\bar{\beta })$ times faster than CRT-RSA(typical RSA) and hashed ElGamal in *G*.

Table 3 shows the practical execution time comparison between parallel scheme and CRT-RSA.

**Table 3:** Practical decryption time comparison between parallel scheme and CRT-RSA (2048-bit modulus).

	Hashed ElGamal in *G* (KEM/DEM)	Parallel scheme (*t* = 2) (KEM/DEM)	Parallel scheme (*t* = 4) (KEM/DEM)	CRT-RSA (OAEP)
Decryption time	15.29ms	8.22ms	4.29ms	15.21ms
Ciphertext overhead	2048bit	4096bit	8192bit	-
Experimental decryption speed up	1	1.86	3.56	1

Timings were made on 3.6GHz Core i7-7700 desktop using Open SSL and can be treated as a relative guideline. We ran the decryption algorithm 1000 times varying keys, each of which included 100 different messages, and obtained the averages. In all measurements, *mod p* and *mod q* exponentiation were done serially and delays by hash function and symmetric encryption were ignored because it is very small compared to modular exponentiation of big integers. As shown in Table 3, our parallel schemes are about 1.86(*t* = 2) and 3.56(*t* = 4) times faster, respectively, than CRT-RSA in decryption, but have the ciphertext overhead increased in proportion to the number of processors.

Overall, the results presented above show that our scheme is suitable to encrypt and decrypt short messages such as session key, credit card information and PIN(Personal Identification Number) code at high speed in multi-core and many-core platforms.

## 5. Discussion

The purpose of converting large private key into the group of small private keys is to reduce the secret exponentiation time by parallel processing. Our technique does not affect the security of original hashed ElGamal, because *r*-bit private key *x*_*p*_(*x*_*q*_) is simply divided into two $\frac{r}{2}$-bit halves *x*_0*p*_ and *x*_1*p*_ (*x*_0*q*_ and *x*_1*q*_) by *h* = 2^⌈*r*/2⌉^.

However, one could reduce the secret exponentiation time further by choosing $h({log}_{n}h\approx 1)$ so that *x*_0*p*_, *x*_1*p*_, *x*_0*q*_ and *x*_1*q*_ are extremely small(i.e., $0<x_{0p},x_{1p},x_{0q},x_{1q}<2^{r\prime }$ and *r*′<*r*/2*)*. In this case, for the security problem, ${log}_{n}x_{0}\approx {log}_{n}x_{1}\approx 1$ must be satisfied for *x*_0_ and *x*_1_ such that $hx_{1}+x_{0}=x\;mod\;\varphi (n)$, $x_{0p}=x_{0}\;mod(p-1),x_{1p}=x_{1}\;mod(p-1),x_{0q}=x_{0}\;mod(q-1)$ and $x_{1q}=x_{1}\;mod(q-1)$. Of course, the time required for encryption is not affected by the selection of *h* because *g*_1_ = *g*^*h*^ is calculated only once in the system. It is an open problem whether there is an attack on parallel scheme when *x*_0*p*_, *x*_1*p*_, *x*_0*q*_ and *x*_1*q*_ are small.

## 6. Conclusion

ICDH assumption is known to be hold only in bilinear group with complex structure. We first proved that ICDH assumption holds in the simple integer group and proposed the CCA secure hashed ElGamal encryption, the security of which is proved in the random oracle model. Our scheme is superior in ciphertext overhead and exponentiation cost to other CCA secure ElGamal variants based on integer group such as Cramer Shoup scheme and twin ElGamal because it maintains the concise style of plain ElGamal. We also sped up decryption of CCA secure hashed ElGamal by parallel processing. Our parallelization scheme does not affect the security since the some operations for decryption have been only pre-calculated at encryption stage and the private key itself is not reduced compared to the hashed ElGamal. By using parallel scheme, it would be possible to use ElGamal in integer group when the big modulus numbers(15360 bit) are used in order to resist quantum computing attack. We expect our finding to be widely applied to the platforms equipped with multicore CPUs or many core GPUs.

## Supporting information

S1 Table Relationship between parameters of Eqs (14) and (15) (r = 1024, t = 2). (DOCX)

S2 Table Relationship between parameters of Eqs (14) and (15) (r = 1024, t = 4). (DOCX)

S3 Table Relationship between parameters of Eqs (14) and (15) (r = 1024, t = 8). (DOCX)

S4 Table Relationship between parameters of Eqs (14) and (15) (r = 1024, t = 16). (DOCX)
