# Supplementary material for: Possibility of decryption speed-up by parallel processing in CCA secure hashed ElGamal
Source: PLoS One. 2023 Nov 30;18(11):e0294840. doi: 10.1371/journal.pone.0294840 (PMC10688657; doi:10.1371/journal.pone.0294840)
Supplement: S1 Table — (DOCX) [file pone.0294840.s001.docx]

**Supporting Information**

In experiment, we use the following left to right exponential algorithm in modular exponentiation.

**Algorithm1. Left to right exponentiation algorithm**Input: $a\in G$ and a positive integer $x={(x_{n}x_{n-1}\cdots x_{2}x_{1})}_{2}$

Output: $a^{x}$

**Step1.** $A=a, S=1, i=n+1.$

**Step2.** $i=i-1,S=S\cdot S$

**Step3.** If $x_{i}=1$ then $S=S\cdot A$

**Step4.** If $i\geq1$ then go to 2.

**Step5.** Return $S$.

In Algorithm1, the number of multiplication is related the Hamming weights of exponents. Some experimental results are as follows.

**TabelB1. Relationship between parameters of Equation (14) and (15)** $\boldsymbol{(r = 1024, t = 2)}$

| No | $r\times0.5$ | $V$ | $W$ | $\left\lceil\frac{r}{t} \right\rceil\times0.5$ | $\max\left\{ V_{i}\vert1\leq i\leq t \right\}$ | $\max\left\{ W_{i}\vert1\leq i\leq t \right\}$ |
| --- | --- | --- | --- | --- | --- | --- |
| 1 | 512 | 520 | 507 | 256 | 277 | 256 |
| 2 | 512 | 516 | 499 | 256 | 268 | 250 |
| 3 | 512 | 508 | 510 | 256 | 269 | 254 |
| 4 | 512 | 498 | 522 | 256 | 248 | 257 |
| 5 | 512 | 491 | 508 | 256 | 251 | 256 |
| 6 | 512 | 525 | 531 | 256 | 264 | 257 |
| 7 | 512 | 515 | 518 | 256 | 261 | 263 |
| 8 | 512 | 513 | 502 | 256 | 249 | 247 |
| 9 | 512 | 508 | 499 | 256 | 261 | 243 |
| 10 | 512 | 511 | 509 | 256 | 252 | 255 |
