# Supplementary material for: Possibility of decryption speed-up by parallel processing in CCA secure hashed ElGamal
Source: PLoS One. 2023 Nov 30;18(11):e0294840. doi: 10.1371/journal.pone.0294840 (PMC10688657; doi:10.1371/journal.pone.0294840)
Supplement: S2 Table — (DOCX) [file pone.0294840.s002.docx]

**Supporting Information**

**TabelB2. Relationship between parameters of Equation (14) and (15)** $\boldsymbol{(r = 1024, t = 4)}$

| No | $r\times0.5$ | $V$ | $W$ | $\left\lceil\frac{r}{t} \right\rceil\times0.5$ | $\max\left\{ V_{i}\vert1\leq i\leq t \right\}$ | $\max\left\{ W_{i}\vert1\leq i\leq t \right\}$ |
| --- | --- | --- | --- | --- | --- | --- |
| 1 | 512 | 499 | 513 | 128 | 123 | 132 |
| 2 | 512 | 536 | 535 | 128 | 143 | 154 |
| 3 | 512 | 516 | 505 | 128 | 134 | 133 |
| 4 | 512 | 502 | 504 | 128 | 130 | 127 |
| 5 | 512 | 529 | 494 | 128 | 137 | 125 |
| 6 | 512 | 498 | 516 | 128 | 120 | 138 |
| 7 | 512 | 499 | 527 | 128 | 138 | 129 |
| 8 | 512 | 518 | 509 | 128 | 142 | 132 |
| 9 | 512 | 512 | 496 | 128 | 144 | 115 |
| 10 | 512 | 501 | 509 | 128 | 134 | 129 |
