# Supplementary material for: Possibility of decryption speed-up by parallel processing in CCA secure hashed ElGamal
Source: PLoS One. 2023 Nov 30;18(11):e0294840. doi: 10.1371/journal.pone.0294840 (PMC10688657; doi:10.1371/journal.pone.0294840)
Supplement: S3 Table — (DOCX) [file pone.0294840.s003.docx]

**Supporting Information**

**TabelB3. Relationship between parameters of Equation (14) and (15)** $\boldsymbol{(r = 1024, t = 8)}$

| No | $r\times0.5$ | $V$ | $W$ | $\left\lceil\frac{r}{t} \right\rceil\times0.5$ | $\max\left\{ V_{i}\vert1\leq i\leq t \right\}$ | $\max\left\{ W_{i}\vert1\leq i\leq t \right\}$ |
| --- | --- | --- | --- | --- | --- | --- |
| 1 | 512 | 491 | 525 | 64 | 75 | 72 |
| 2 | 512 | 495 | 520 | 64 | 72 | 75 |
| 3 | 512 | 513 | 499 | 64 | 73 | 64 |
| 4 | 512 | 490 | 518 | 64 | 68 | 68 |
| 5 | 512 | 517 | 551 | 64 | 71 | 72 |
| 6 | 512 | 514 | 519 | 64 | 78 | 70 |
| 7 | 512 | 506 | 504 | 64 | 75 | 68 |
| 8 | 512 | 504 | 508 | 64 | 70 | 75 |
| 9 | 512 | 517 | 497 | 64 | 72 | 68 |
| 10 | 512 | 498 | 509 | 64 | 70 | 65 |
