# Supplementary material for: Possibility of decryption speed-up by parallel processing in CCA secure hashed ElGamal
Source: PLoS One. 2023 Nov 30;18(11):e0294840. doi: 10.1371/journal.pone.0294840 (PMC10688657; doi:10.1371/journal.pone.0294840)
Supplement: S4 Table — (DOCX) [file pone.0294840.s004.docx]

**Supporting Information**

**TabelB4. Relationship between parameters of Equation (14) and (15)** $\boldsymbol{(r = 1024, t = 16)}$

| No | $r\times0.5$ | $V$ | $W$ | $\left\lceil\frac{r}{t} \right\rceil\times0.5$ | $\max\left\{ V_{i}\vert1\leq i\leq t \right\}$ | $\max\left\{ W_{i}\vert1\leq i\leq t \right\}$ |
| --- | --- | --- | --- | --- | --- | --- |
| 1 | 512 | 496 | 533 | 32 | 37 | 39 |
| 2 | 512 | 527 | 486 | 32 | 38 | 36 |
| 3 | 512 | 519 | 514 | 32 | 44 | 38 |
| 4 | 512 | 501 | 504 | 32 | 40 | 40 |
| 5 | 512 | 522 | 501 | 32 | 40 | 46 |
| 6 | 512 | 507 | 522 | 32 | 38 | 40 |
| 7 | 512 | 533 | 493 | 32 | 41 | 36 |
| 8 | 512 | 520 | 511 | 32 | 40 | 36 |
| 9 | 512 | 538 | 492 | 32 | 41 | 41 |
| 10 | 512 | 534 | 517 | 32 | 41 | 38 |

As shown in above tables, $V$ and $W$ are similar to $r\times0.5$, but $\max\left\{ V_{i}|1\leq i\leq t \right\}$ and $\max\left\{ V_{i}|1\leq i\leq t \right\}$ are usually larger than $\left\lceil\frac{r}{t} \right\rceil\times0.5$.
